# Hydroxytyrosol, a Promising Supplement in the Management of Human Stroke: An Exploratory Study

**DOI:** 10.3390/ijms25094799

**Published:** 2024-04-27

**Authors:** Ángela Naranjo, M. Josefa Álvarez-Soria, Pilar Aranda-Villalobos, Ana M. Martínez-Rodríguez, Esther Martínez-Lara, Eva Siles

**Affiliations:** 1 Departamento de Biología Experimental, Universidad de Jaén, 23071 Jaén, Spain; anaranjo@ujaen.es; 2 Servicio de Neurología, Hospital Universitario de Jaén, 23009 Jaén, Spain; mjosefa.alvarez.sspa@juntadeandalucia.es; 3 UGC Medicina Física y Rehabilitación, Hospital Universitario de Jaén, 23009 Jaén, Spain; pilar.aranda.villalobos@gmail.com; 4 Departamento de Estadística e Investigación Operativa, Universidad de Jaén, 23071 Jaén, Spain; ammartin@ujaen.es

**Keywords:** ischemic stroke, hydroxytyrosol, oxidative-stress-related markers, lipid profile, blood pressure, HbA₁c, proteomics, neurological and functional outcomes

## Abstract

Hydroxytyrosol (HT) is a bioactive olive oil phenol with beneficial effects in a number of pathological situations. We have previously demonstrated that an HT-enriched diet could serve as a beneficial therapeutic approach to attenuate ischemic-stroke-associated damage in mice. Our exploratory pilot study examined this effect in humans. Particularly, a nutritional supplement containing 15 mg of HT/day was administered to patients 24 h after the onset of stroke, for 45 days. Biochemical and oxidative-stress-related parameters, blood pressure levels, serum proteome, and neurological and functional outcomes were evaluated at 45 and 90 days and compared to a control group. The main findings were that the daily administration of HT after stroke could: (i) favor the decrease in the percentage of glycated hemoglobin and diastolic blood pressure, (ii) control the increase in nitric oxide and exert a plausible protective effect in oxidative stress, (iii) modulate the evolution of the serum proteome and, particularly, the expression of apolipoproteins, and (iv) be beneficial for certain neurological and functional outcomes. Although a larger trial is necessary, this study suggests that HT could be a beneficial nutritional complement in the management of human stroke.

## 1. Introduction

Stroke is the second leading cause of death and the third leading cause of death and disability combined worldwide [1]. In Spain, according to data extracted from the Annual Report of the National Health System 2020–2021, it affects 1.5% of the population [2]. The aging of the population exerts a negative impact on its prevalence, and 25% of people over age 25 will suffer a stroke in their lifetime. It should be noted that between the two most common types, ischemic and hemorrhagic, the first represents 80–85% of the total, and advances in primary prevention are unable to control the growing number of cases [1].

The ineffectiveness of current treatments is a notable problem. One of the most commonly used therapies is based on the dissolution of the thrombus through treatment with a tissue plasminogen activator. However, it can only be administered within the first few hours and in many cases is not useful. Moreover, it does not prevent neurodegeneration after stroke and is associated with a high number of complications, such as hemorrhagic transformation [3]. Likewise, the second preferred option is the so-called endovascular therapy, but it shares many of the limitations of the previous one [4]. These, along with other therapies that are somewhat less common, are insufficient, not only because of their success rate but also because the approach must be directed toward protection against reperfusion and promotion of neuronal regeneration.

Non-modifiable risk factors associated with ischemic stroke are age, sex, and genetics. The modifiable ones, which represent 91.5% of the risk of suffering a cardiovascular accident of this type, include a history of hypertension or blood pressure problems, low physical activity, high ratios of apolipoprotein (Apo) B and ApoA, hyperglycemia, hip–waist correlation, psychosocial factors, stress, depression, smoking, high alcohol consumption, and diet [5,6]. As for the latter, it has been shown for years that good nutrition is essential for cardiovascular diseases. In fact, following a poor diet can be related to 31% of strokes [1]. For more than 50 years, a decreased rate of coronary heart disease and ischemic stroke has been detected in the Mediterranean area [7]. Mediterranean diet (MD) patterns can be slightly different depending on the geographic location but the main characteristic they all share is the consumption of extra virgin olive oil (EVOO). This oil is mainly composed of fatty acids, but its particular mechanical extraction at low temperatures also affords a high concentration of different minor components, such as phenols. The main phenolic alcohol is hydroxytyrosol (HT), which has beneficial effects in a multitude of diseases associated, among others, with its antioxidant and anti-inflammatory properties [8]. The former is related to its ability to neutralize free radicals through hydrogen donation [9], and to the activation of different cellular signaling pathways [10] that in the end can, for example, modulate the production of nitric oxide (NO) or the expression of antioxidant enzymes.

In an ischemic stroke, the lack of blood flow leads to obvious cellular damage. The smaller amount of blood in the bloodstream reduces the availability of glucose and oxygen and, therefore, there is a lower energy supply to neurons, endothelial cells, and glia. Cellular damage is also linked to an increase in the concentration of glutamate outside the cells and to a neuronal calcium influx [11]. As a whole, this ischemic cascade triggers mitochondrial alterations, protein misfolding, astrocytic changes, and an inflammatory response due to dysfunction in the blood–brain barrier with the consequent release of signaling molecules (e.g., cytokines) and the increase in free radicals [12]. Therefore, it is evident that HT, due to its anti-inflammatory and antioxidant action, could be useful to counteract the ischemic-cascade-associated damage.

Until now, most studies analyzing the neuroprotective action of HT in ischemia have been carried out using ex vivo ischemia models with doses of HT between 1 and 20 mg/kg/day. The results obtained indicate that HT exerts a neuroprotective effect related to a decrease in nitrosative and oxidative stress, to a lower release of lactate dehydrogenase and to a reduction in inflammatory processes [13,14,15,16,17]. Besides, our research group carried out two preclinical studies to evaluate the therapeutic effect of a diet supplemented with 0.03% HT in mice that had suffered a stroke using the transient middle cerebral artery occlusion model. Through a multidisciplinary approach, we showed that the supplementation with HT improved non-associative learning and motor abilities, being a promising compound to reduce cognitive deficits associated with stroke. Using different MRI techniques, we found that it also improved functional and structural connectivity between various brain regions in the infarcted animals and increases cerebral blood flow. Moreover, the HT-enriched diet induced an anti-inflammatory response along with a greater expression of the synaptic-neurogenic marker Psd-95. This phenol also increased muscle strength recovery within the first two weeks [18,19]. In short, evidence points to HT as a promising compound to improve the clinical status of patients after a stroke.

To gain insight into the overall picture underlying a particular disease, the use of omics is becoming increasingly important in the clinical setting, as they offer relevant information about the molecular pathways involved. Particularly, proteomics has proven to be very useful for evaluating the severity of ischemic damage and its evolution with easily obtained samples such as blood [20,21]. Therefore, this technique can be complementary to other approaches when analyzing the therapeutic effect of a particular compound.

With this background, and by using biochemical and proteomic analysis together with the assessment of neurological and functional outcomes, we have carried out an exploratory pilot study to evaluate the effect of HT in the management of human stroke.

## 2. Results

### 2.1. Biochemical and Oxidative-Stress-Related Parameters

**Figure 1:**
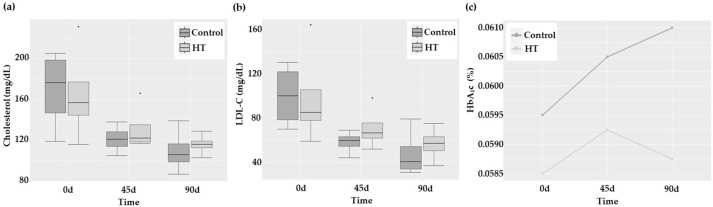
(**a**,**b**) Boxplots presenting median and quartiles of TC and LDL-C values, respectively, and (**c**) profile plot of HbA_1_c in control and HT-treated stroke patients at 0, 45, and 90 days. * *p*-value = 0.034.

Biochemical parameters of the control and HT groups are presented in Table 1. Glucose, triglycerides (TAG), and high-density lipoprotein cholesterol (HDL-C) levels showed no significant changes with time. Besides, no group effect was detected. Conversely, total cholesterol (TC; Figure 1a) showed a marginal decrease with time independently of the group (*p* = 0.070), probably linked with the time-dependent significant reduction in low-density lipoprotein cholesterol (LDL-C; *p* = 0.008). The percentage of glycated hemoglobin (HbA_1_c) did not show any statistically significant differences; however, the profile plot in Figure 1c indicated a trend to decrease at 90 days only in the HT group.

**Figure 2:**
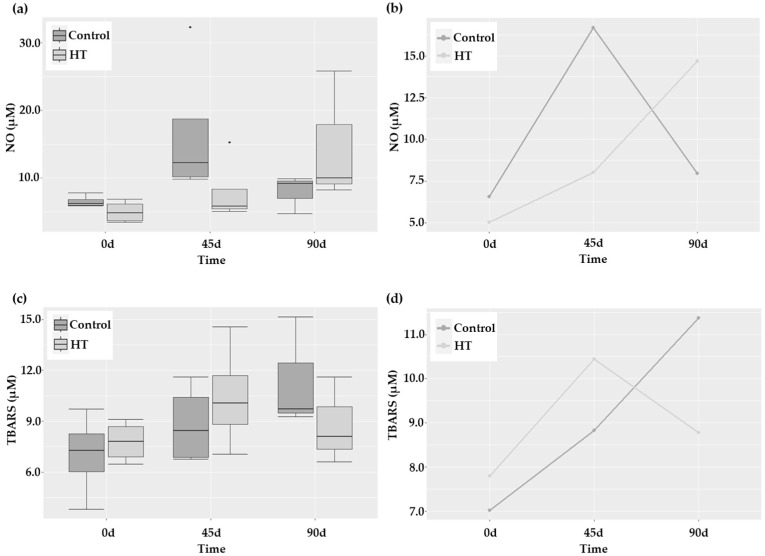
Oxidative-stress-related parameters at 0, 45, and 90 days after stroke in control and HT-treated patients. (**a**,**c**) Boxplots showing median and quartiles and (**b**,**d**) profile plots of the distribution of NO and TBARS levels. * *p*-value = 0.034.

Interleukin 6 (IL-6) is a marker of systemic inflammation that has been associated with functional outcome after stroke [22]. In this study, IL-6 levels showed a trend to decrease (*p* = 0.08; Table 1) with no group-associated differences. However, the analysis of NO, another biomarker of inflammation linked with endothelial dysfunction, revealed a different behavior in both groups (*p* = 0.01) that was corroborated by the profile plot (Figure 2a,b). Particularly, the increase in NO at 45 days was sharper in control patients than in those receiving HT. Nevertheless, in the latter, the levels increased after suspending HT treatment, although this increase was due to a particular patient. Finally, TBARS (thiobarbituric acid-reactive substances) levels, indicative of lipid peroxidation, did not exhibit a different pattern in response to HT, although a marginally significant time effect was observed (*p* = 0.063; Figure 2c). Even though no group effect could be statistically demonstrated, the different and non-parallel pattern observed in the profile plot of the estimated marginal means of this parameter (Figure 2d) seems to indicate a plausible protective effect of HT in the long term.

### 2.2. Effect of HT on Blood Pressure

**Figure 3:**
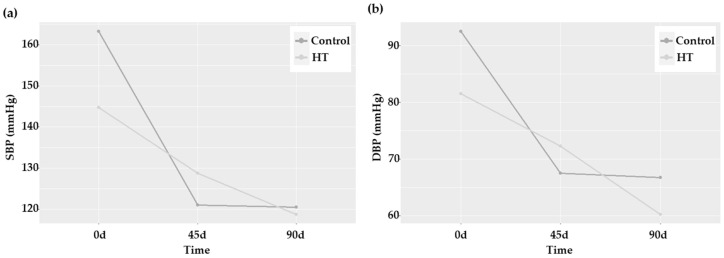
Blood pressure profile plots of patients 0, 45, and 90 days after stroke in control and HT-treated patients. (**a**) SBP and (**b**) DBP.

High blood pressure (BP) levels are associated with an increased incidence of stroke and worse functional outcomes [23]. Therefore, we wondered whether HT would be able to decrease systolic and diastolic BP (SBP and DBP, respectively). As shown in Table 1, the values of these two parameters significantly changed with time (*p* = 0.004 for SBB, and *p* = 0.001 for DBP), but this effect was achieved in both groups without any statistically significant difference between them. Nevertheless, the crossing of the lines when comparing the estimated marginal means in the profile plots was indicative of an HT-induced differential response (Figure 3). Particularly, DBP showed a more prolonged decrease in patients treated with HT.

### 2.3. Effect of HT on Serum Proteome

**Figure 4:**
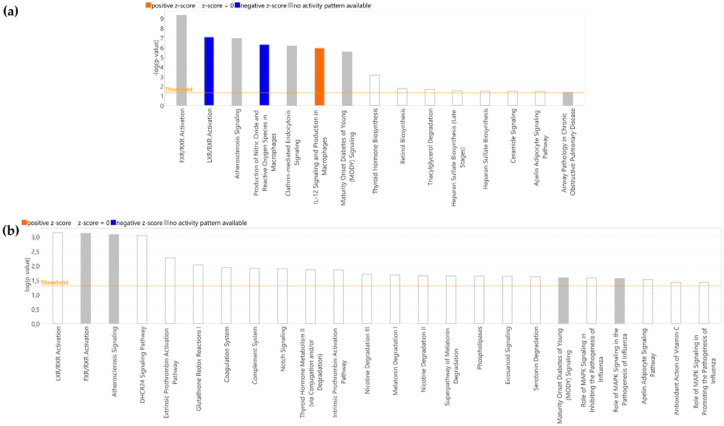
Canonical pathways modified in (**a**) HT and (**b**) control groups at 45 versus 0 days. Bars in orange (upregulated) and blue (downregulated) indicate z-score values greater or lower than 2. Pathways with a −log (*p*-value) over 1.3 are shown.

The modulatory effect of HT in the evolution of the serum proteome of stroke patients was assessed by Nano-LC-MS/MS. A total of 339 proteins were identified: 9 of these proteins were found to be differentially expressed 45 days after stroke in HT-treated patients, 2 significantly overexpressed, and 7 under-expressed (Table 2). A similar number of differentially expressed proteins (DEP) were found in the control group, with five significantly overexpressed and three under-expressed, although none of them were common to the ones observed in the HT-treated group. Then, we analyzed the molecular pathways associated with these DEPs and found 15 enriched canonical pathways in HT-treated patients (Figure 4a) and 24 in control patients (Figure 4b). Among these, the top three according to the *p*-value were common to both groups: Farnesoid-X Receptor/Retinoid-X Receptor (FXR/RXR) activation, Liver-X Receptor/Retinoid-X Receptor (LXR/RXR) activation, and Atherosclerosis signaling. Although none of the enriched canonical pathways in control patients showed a significant z-score, in the HT group, LXR/RXR activation and production of nitric oxide and reactive oxygen species in macrophages showed a z-score = −2, and IL-12 signaling and production in macrophages a z-score = 2. These significant z-scores were mainly due to the decreased expression of ApoB100, ApoE, ApoM, and prenylcysteine oxidase I (PCYOX1). Although not involved in these pathways, lipase E (LIPS; more commonly known as hormone-sensitive lipase, HSL) was also drastically decreased (45 d/0 d fold-change = −3.229) in stroke patients after 45 days of HT intake.

When we analyzed the changes in the proteome of patients 90 days after stroke versus 45 days (Table 2), only 2 proteins, K2C78 and TTR, were significantly overexpressed in the HT group. However, seven DEP were identified in the control group (five under-expressed: ANK1, FA9, B4GA1, SAA4, and CROCC, and two overexpressed: PSA7 and CD109). None of those changes involved the appearance of canonical pathways with a significant z-score value.

**Figure 5:**
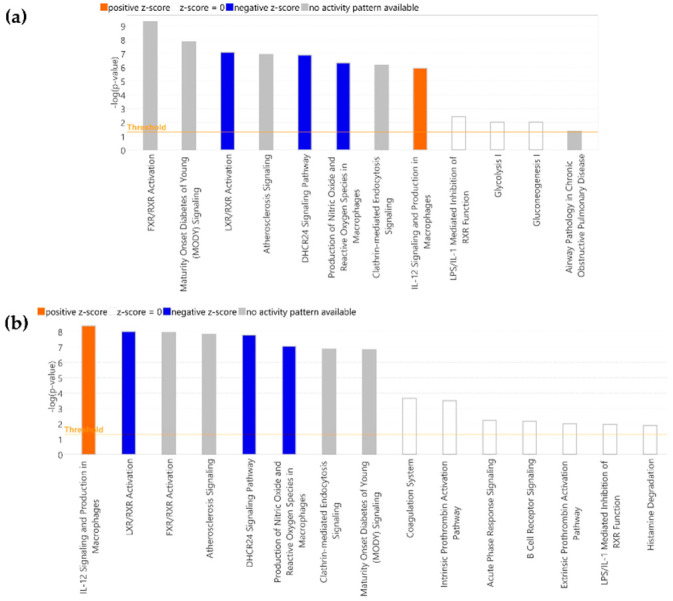
Canonical pathways modified in (**a**) HT and (**b**) control groups at 90 versus 0 days. Bars in orange (upregulated) and blue (downregulated) indicate z-score values greater or lower than 2. Top 15 pathways with a −log (*p*-value) over 1.3 are shown.

Finally, when we compared the evolution of the proteome from the beginning to the end of the study (Table 2, 90 d/0 d), we found nine DEPs in the HT group (four under-regulated: ApoC4, PGK1, ApoC1, and ApoM, and five upregulated: ApoA4, QPCT, K1C14, HABP2, and FETUB), and fifteen DEPS in the control group (nine under-expressed: KV108, IGJ, SAA4, ApoM, FA9, FUCO, ApoC1, FHR1, and ApoA2, and six upregulated: ApoA4, Ant3, CD109, CNTN1, AL1A1, and KV37). In HT-treated patients, these DEPs were associated with changes in 12 canonical pathways (Figure 5a), 4 of them with significant z-scores (LXR/RXR activation, DHCR24 signaling pathway, production of nitric oxide and reactive oxygen species in macrophages, and IL-12 signaling and production in macrophages). In the control group, there were significant changes in 34 canonical pathways (the top 15 are shown in Figure 5b), with the first 8 being according to the *p*-value and another 4 with a significant z-score in common with the HT group.

### 2.4. Effect of HT on Neurological and Functional Outcomes after Stroke

The evolution of the severity of neurological deficits in both groups of stroke patients (control and HT-treated) is shown in Table 1.

**Figure 6:**
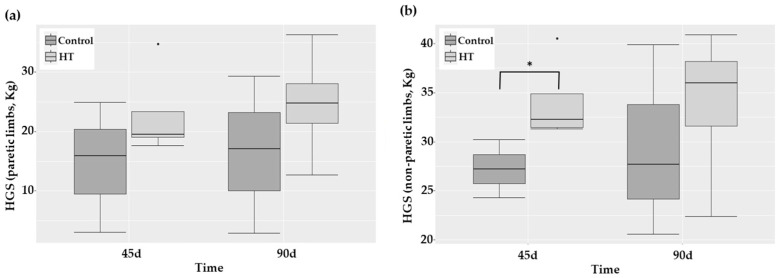
Boxplots showing median and quartiles of the HGS of (**a**) paretic limbs and (**b**) non-paretic limbs in patients 0, 45, and 90 days after stroke. * *p*-value = 0.034.

The follow-up of the National Institutes of Health Stroke Scale (NIHSS) and Rankin Scale (mRS) score indicated a parallel decrease with time in both groups (*p* = 0.001 and *p* < 0.001, respectively), particularly sharp from 0 to 45 days. Although no statistically significant overall effect of HT treatment was detected, stroke HT-treated patients exhibited a lower mRS score than control ones (*p* = 0.034) at 45 days after. The evaluations of risk of falling (Timed Up and Go, TUG), walking functionality (Functional Ambulation Category, FAC), muscle strength (hand-grip strength, HGS), and cognitive dysfunction (Montreal Cognitive Assessment test, MoCA score) were similar along time and in both groups, with the only exception being HGS of non-paretic limbs. This latter parameter was significantly higher in HT-treated patients than in the control group 45 days after stroke (Figure 6; *p* = 0.034).

## 3. Discussion

HT is a bioactive olive oil phenol whose positive effects in a number of pathological situations have been extensively described in the literature. In an in vivo study with mice, we have previously demonstrated that an HT-enriched diet could serve as a beneficial therapeutic approach to attenuate ischemic-stroke-associated damage [18,19]. The present pilot study examined this effect in humans. The main findings were that the daily administration of HT after stroke could: (i) favor the decrease in the percentage of HbA_1_c and DBP, (ii) modulate the increase in NO and exert a plausible protective effect in oxidative stress, (iii) change the evolution of the serum proteome and, particularly, the expression of apolipoproteins, and (iv) be beneficial for certain neurological and functional outcomes.

Hyperglycemia has been associated with a higher risk of ischemic stroke, particularly in larger-artery and small-vessel stroke, and HbA_1_clevels rather than glucose are predictors of worse outcomes following endovascular thrombectomy [6,24]. Therefore, any therapeutic approach able to lower HbA_1_cwould contribute to decreasing the risk of ischemic stroke. The positive effect of HT on glycemic control has already been evaluated in a number of studies. Santangelo et al. [25] observed that the intake of extra virgin olive oil with a high polyphenol content reduced basal blood glucose, HbA_1_c, and body mass index (BMI) in patients with type 2 diabetes. More recently, it has also been published that daily consumption of bread enriched in HT (54 mg HT/100 g of bread; 32.5 mg HT/day) causes a significant reduction in body fat mass and positive effects on fasting glucose levels, HbA_1_c, and insulin, as well as reducing inflammatory markers and blood lipid levels [26]. Our present results indicate that the percentage of HbA_1_c showed a trend to decrease at 90 days only in the HT group, suggesting that the administration of this phenol after a stroke would be particularly beneficial for patients.

Blood pressure is another crucial factor in cardiovascular outcomes. Although systolic blood pressure is considered to be more important than diastolic blood pressure in the determination of cardiovascular risk, Flit et al. [27] found that systolic and diastolic hypertension independently predicted adverse outcomes, despite a greater effect of systolic hypertension. In two recent papers, Ikobomidies et al. [28] and Hara et al. [29] described that an HT-enriched olive oil and a 6% HT-enriched diet reduced the oxidative and inflammatory burden in chronic coronary artery syndrome patients and in apolipoprotein E-deficient mice, but had no effect on BP. In our study, HT did not modulate SBP, but seemed to induce a more prolonged decrease in DBP, resembling the pattern of response of HbA_1_c and reinforcing the positive effect of HT.

Atherosclerosis, a major cause of stroke in humans, is a chronic inflammatory disease that is characterized by intimal plaques and cholesterol accumulation in the arterial walls [30]. Oxidative stress, produced by the imbalance between reactive oxygen and/or nitrogen species’ formation and the antioxidant defense system, is the main cause of stroke damage. NO is a free radical, which acts as a neuromodulator in the central nervous system, being involved in the maintenance of the vascular tone and brain microcirculation, neurotransmission, and epigenetic regulation [31]. In fact, it modulates long-term synaptic transmission and promotes synaptogenesis and synaptic remodeling. NO functions are deeply regulated by its concentration. A moderate increase in NO levels is beneficial; however, large increases, particularly in a pro-oxidant environment, are detrimental [32]. In this sense, we previously demonstrated that, after an ischemic stroke, the time at which the elevation of NO occurs is linked to the outcome [33]. Thus, the initial decrease in NO levels is followed by a progressive upregulation, in such a way that the increase from day 1 to 2 is beneficial to patients, but a later steep increase is detrimental. HT is a well-known antioxidant and a modulator of NO production [9,34] with an atheroprotective role achieved, among others, by a decreased expression of the inducible isoform of NO synthase (iNOS) and by the downregulation of oxidative stress [28,29]. In this study, we showed that the administration of HT modulated NO production (*p* = 0.001). The increase in NO, observed in both groups, was much more prominent in control patients than in those receiving HT. This result could be linked to a higher activation of the inducible isoform of NO synthase and, therefore, to a detrimental pro-inflammatory signature counteracted by HT. Strikingly, NO levels increased after suspending HT treatment (90 d), although this increase was due to a particular patient. Finally, HT did not seem to exert any statistically significant effect on oxidative stress, but the different and non-parallel pattern observed in the profile plot of the estimated marginal means of TBARS seemed to indicate a plausible protective effect of HT in the long term. These results would be in agreement with the more prolonged decrease in DBP observed in the HT group. A future study with a larger number of patients is necessary to confirm these results.

Proteomics, by analyzing the complete protein landscape in a particular sample, is a very valuable tool to gain insight into the precise molecular mechanism underlying a biological situation. The effect of stroke on human plasma proteome has already been analyzed [20]. In the present study, we comparatively examined the evolution of the serum proteome in HT and control groups. We demonstrated that the intake of HT is associated with significant changes in the evolution of the serum proteome of stroke patients. Among the 9 DEP observed in HT-treated patients 45 days after stroke, the downregulation in ApoB100, ApoE, ApoM, and PCYOX1 was remarkable, as all these proteins are involved in the alteration of most of the canonical pathways observed in Figure 4. Lipids are known to exert a crucial role in ischemic stroke, but the focus, traditionally placed on cholesterol and triacylglycerides, has nowadays moved to apolipoproteins. ApoB100 can be found in atherogenic lipoproteins that became trapped in the arterial wall, producing atherosclerosis. A number of studies have demonstrated that the increase in the level of this apolipoprotein rather than in the amount of cholesterol and triacylglycerides is crucial to promote ischemic stroke [35]. PCYOX1 is another crucial player in atherogenesis. It is highly expressed by macrophages, endothelial and smooth muscle cells (SMC), and transported within the subintimal space by ApoB100-containing lipoproteins. Once there, PCYOX1 reacts with prenylcysteine to produce oxidant species that promote the oxidation of ApoB100-containing lipoproteins and the development of the atherosclerotic lesion [36]. With this background, the decrease in ApoB and PCYOX1 observed in HT-treated stroke patients could be considered as indicative of an atheroprotective effect of HT. The proteome of HDL lipoproteins also suffers an active remodeling after stroke. In fact, changes in HDL proteins during the early acute phase of stroke have been shown to be associated with recovery [37]. Particularly, the increase in ApoE and ApoM 24 and 96 h post-stroke has been related to a worse three-month recovery score, as assessed by the NIHSS score. Similarly, a high HDL-ApoE concentration has also been linked to a higher risk of coronary heart disease events due, in part, to the increased production of VLDL (very-low-density lipoprotein) and lower VLDL lipolysis by lipoprotein lipase [38]. Therefore, the downregulation of ApoE and ApoM induced by HT could be considered as indicators of a better recovery. In fact, and in agreement with this suggestion, we have shown that 45 days after stroke, HT patients exhibited a lower mRS score than control stroke patients (*p* = 0.034) and a higher HGS of non-paretic limbs (*p* = 0.034). Altogether, these results reinforce that the intake of HT could be a promising strategy after stroke. HT intake also decreased the expression of HSL, an enzyme abundant in adipose tissue that hydrolyzes diacylglycerols, generating monoacylglycerols and fatty acids. A recent metabolomic analysis carried out in serum revealed a consistent increase of mono/diacylglycerols and medium/long-chain fatty acids in the acute phase of ischemic stroke [39], suggesting an increased activation of this enzyme. HSL also has a cholesteryl-ester hydrolase activity, and its expression in macrophages favors cholesterol efflux from foam cells. However, some studies have described that macrophage-specific transgenic expression of HSL resulted in more advanced atherosclerosis. This effect could not be explained by altered lipid plasma levels and, in turn, the authors pointed to its indirect effects on inflammation [40]. In such case, HT-mediated downregulation of this enzyme would be in agreement with a lower inflammatory state, consistent with the inhibition in the canonical pathway “production of nitric oxide and reactive oxygen species in macrophages” observed after the intake of HT in stroke patients (Figure 4). Moreover, this result would also agree with the attenuated inflammatory activation of macrophages and the reduced vascular inflammation recently described by Hara et al. [29] in HT-treated mice. These data contrast with those observed in control patients. The only apolipoprotein differentially expressed in this case was ApoA4, which was significantly upregulated. This apolipoprotein is present on chylomicron remnants, HDL lipoproteins, and in lipid-free form. ApoA4 is involved, among others, in lipid metabolism, reverse cholesterol transport, glucose metabolism, and protection against atherosclerosis, platelet aggregation, and thrombosis [41]. The research of Plubell et al. [37] described that although it is differentially expressed after stroke, its levels cannot be correlated with stroke recovery scores. Similarly, the relationship between serum levels of contactin-1 (CNTN1), a cellular adhesion molecule involved in axo–glial interaction, and stroke is not well defined, and even the literature is controversial [42,43]. Its role in mouse brain tissue has been somewhat more studied, where it seems to have a pro-inflammatory role by increasing the expression of IL-6 [44]. Therefore, the absence of this protein in the HT group would again suggest a positive effect of this phenol. In fact, lecithin cholesterol acyl transferase (LCAT) and glutathione peroxidase 3 (GPX3) were downregulated in control patients but not in HT-treated patients. The downregulation of LCAT after stroke has already been described in the literature. This enzyme esterifies cholesterol in HDL and hydrolyzes the acyl group at the sn-2 position of HDL phospholipids. Therefore, it is associated with HDL atheroprotective functions, including cholesterol efflux capacity, and its downregulation is linked with the severity and outcome of acute ischemic stroke [45]. GPX3 is an antioxidant enzyme, associated with HDL particles, that scavenges reactive oxygen species in the extracellular compartment. The deficiency of this enzyme has been linked with platelet-dependent thrombosis, and contrary to what happened in the HT group, would indicate the existence of an oxidative and prothrombotic state, that would promote platelet-dependent arterial thrombosis [46]. However, the increase in Antithrombin III, not observed in the HT group, was positive, as it inhibited the thrombosis [47]. Finally, beta-1,4-glucuronyltransferase 1 (B4GA1) and Tissue α-L-Fucosidase (FUCO) were also up- and down-regulated, respectively, in control patients. Glycosylation, the pathway in which these two enzymes are involved, is the most abundant and diverse post-translational modification of proteins. B4GA1 is a xylopyranoside β1,4-glucuronyltransferase [48] and α-L-Fucosidase is a soluble lysosomal enzyme that hydrolyzes α-l-fucose residues linked to the 2 position of galactose or the 3, 4, or 6 position of N-acetylglucosamine. The abnormal expression levels of glycosyltransferases and glycosidases have been linked to inflammation and neurodegeneration [49]. Thus, again, the presence of these two DEPs in the control group but not in the HT-treated one would be indicative of the protective effect of this phenol.

Despite the prominent changes described above, when we compared the serum proteome of patients 90 days after stroke versus 45 days, only Transthyretin (TTR) and Keratin 78 (K2C78) were found to be differentially expressed, and particularly upregulated, in the HT group. This result contrasts with the change in seven proteins (five downregulated and two upregulated) observed in control patients and suggests a long-lasting effect of HT, as the intake of this phenol had already been suspended in this period of time. TTR is a protein involved in retinol transport, typically used for assessing the nutritional status. Its expression is downregulated by IL-6 and other inflammatory mediators and, therefore, it is a negative acute phase protein. Lower levels of this protein correlate with poor prognosis in stroke patients [50,51,52,53] and promote carotid intima-media thickening, increasing the risk of recurrence in patients with ischemic stroke [54]. Although in our study we found no group-associated differences in IL-6 levels, the profile plot of the estimated marginal means of TBARS levels points to a plausible protective effect of HT in the long term that could be linked with the upregulation of Transthyretin. Therefore, these results are consistent with a lower inflammatory status at 90 days in HT-treated stroke patients and point to a better outcome. Keratins are involved in keratinization but also determine the immune and inflammatory state. Particularly, the expression of Keratin 78 has been negatively correlated with inflammation and with the infiltration of macrophages in head and neck squamous cell carcinoma and in eosinophilic esophagitis [55]. Therefore, the increase in Keratin 78 points again to an anti-inflammatory effect of HT, beneficial to improve the recovery from stroke and to minimize its recurrence.

Finally, when we analyzed changes in both groups from the beginning to the end of the study (90 days versus 0 days), we observed striking similarities in the DEPs, particularly in apolipoproteins, and in the associated canonical pathways. In both cases, there was an increase in ApoA4 and a decrease in ApoM and ApoCI. However, a downregulation of ApoC4 and ApoA2 was detected in HT-treated and control patients, respectively. According to the literature, ApoC4 levels significantly correlate with NIHSS scores three months after stroke. Thus, the downregulation of this apolipoprotein in the HT group should be an indicator of a better outcome. However, the impact of ApoA2 in cardiovascular disease is controversial. This apolipoprotein is the second most abundant in HDL and the most lipophilic of the exchangeable apolipoproteins. It has been suggested to have anti-atherogenic and pro-atherogenic properties. This would explain why some studies showed that its downregulation is related with a higher incidence of coronary heart disease [56] and stroke [57], while others pointed to a pro-atherogenic activity achieved, among others, by increased LDL-oxidation [58]. Therefore, it is not easy to discuss the effect of its change. Finally, the canonical pathways modulated in both groups were partially similar. Nine out of the twelve pathways observed in the HT group were common to the control group, among them those with a significant z-score. However, a number of pathways related to inflammation, e.g., the coagulation system, intrinsic prothrombin activation, acute phase signaling, or extrinsic prothrombin activation pathways, only appeared in the control group. This result would again be in agreement with a long-term anti-inflammatory action of HT, particularly important after stroke.

As a whole, the biochemical and proteomic analysis presented in this exploratory pilot study, together with the neurological and functional outcomes evaluated, showed that administering HT after stroke could be a promising complementary strategy in the management of human stroke, whose feasibility is worthy of study in a large-scale cohort of stroke patients.

### Limitations

Although the results presented in this study point to the benefits of administering HT after stroke, the sample size was very small. Therefore, the results of this pilot study must be confirmed in a lager sample size to further assess this effect.

## 4. Materials and Methods

### 4.1. Research Design

In this randomized, controlled, double-blind pilot study, 8 individuals with minor or moderate severe acute ischemic stroke, and without dysphagia, were randomly allocated in the 24 h post-stroke to a 45-day treatment with a daily nutritional supplement (Mediteanox^®^) containing 15 mg of HT (HT group) or placebo (control group). Mediteanox or placebo were provided in coded bottles, masked for both patients and researchers.

### 4.2. Subjects

Participants were recruited from the patients hospitalized in the Stroke Unit of the Neurology Service of the Complejo Hospitalario of Jaén from February to May 2022. Subjects selected were screened for eligibility, and informed consent was obtained from each participant. Specific inclusion criteria for participation in this study were men and women with acute ischemic stroke confirmed by MR/CT (magnetic resonance/computed tomography), NIHSS score < 24 and >4, pre-stroke modified mRS ≤ 3, time between initial symptom and admission < 24 h, and absence of dysphagia. Exclusion criteria were age less than 18 years, cerebral hemorrhage, stroke not diagnosed by MR/CT, dysphagia, temperature > 38 °C or infection on hospital admission, advanced renal failure (MDRD < 30), and life expectancy < 3 months.

### 4.3. Data Collection and Sampling

After enrollment in the study, sociodemographic, anthropometric (BMI), and clinical data (medical history, cardiovascular risk factors, and medication prior to admission), as well as blood pressure, were collected.

Peripheral venous blood samples were collected upon admission (in the 24 h after the onset of stroke, initial time of treatment, 0 d), at 45 days (last time of treatment, 45 d), and 30 days later (90 d). Plasma and serum were immediately obtained after centrifugation, analyzed by routine methods in the Laboratory of Analysis of the Complejo Hospitalario of Jaén, aliquoted, and stored at −80 °C until proteomic and oxidative-stress-related analysis.

### 4.4. Evaluation of Neurological and Functional Outcomes

The severity of stroke and disability were evaluated by NIHSS and mRS, respectively, at admission, the last day of treatment (45 d), and 30 days later (90). Functional outcomes of participants were also assessed by measuring the risk of falling (TUG), walking functionality (FAC), muscle strength (HGS, by hand-held dynamometer), and cognitive dysfunction (MoCA score), 45 and 90 days post-stroke.

The NIHSS score is defined as the sum of 15 individually evaluated elements, and ranges from 0 to 42. Stroke severity is categorized as follows: no stroke symptoms, 0; minor stroke, 1–4; moderate stroke, 5–15; moderate to severe stroke, 16–20; severe stroke, 21–42 [59,60]. This tool was validated and translated into Spanish [61].

The mRS scale allows to categorize the level of functional independence with reference to pre-stroke activities. The scale comprises seven levels, from 0 to 6, with 0 to 1 indicating no disability, 2 to 5 indicating increasing disability, and 6 indicating death [62].

The TUG test was used as an easily assessable, general measure of physical function. In the TUG test, participants stand up from a chair (preferably without using the arms), walk 3 m (marked by a tape), turn, and return to the chair to sit down again as quickly and as safely as possible. The time taken to complete the test was recorded and rounded to whole seconds. Participants were allowed to use their walking aid and to use the armrest for support when getting up. Performance of the TUG is rated on a scale from 1 to 20 s, where <10 indicates normal function, 10 to 20 indicates a risk of falling, and >20 indicates a high risk of falling [63,64].

The FAC scale distinguishes six levels of walking ability based on the amount of physical support required, with scores ranging from 0 (non-functional ambulation) to 5 (independent ambulation on any surface). This easily allows categorization of a patient’s level of ambulation [65,66,67].

HGS of the paretic and non-paretic limb was evaluated using a hydraulic hand-grip dynamometer (Kern Map Version 1.2 08/2012), which has demonstrated high levels of reliability for individuals with chronic stroke. The participants were made to sit on a straight-backed armless chair with their feet flat on the floor, elbow flexed at 90°, and the dynamometer was held by the testing hand in a neutral grip without support, in line with the protocols of the American Society of Hand Therapists. The mean value of three trials, with 60 s rest between each trial, was recorded in kilograms. The non-paretic UL was always tested first [68,69,70,71].

Cognitive function was assessed with the MoCA, a cognitive screening tool with high sensitivity and specificity for detecting mild cognitive impairment. The score range is 0 to 30, and the threshold for normal cognitive function is ≥26. The MoCA has eight cognitive domains: executive and visuospatial function, naming, short-term memory, attention, language, abstraction, delayed recall, and orientation to time and space [72]. This tool was validated and translated into Spanish [73].

### 4.5. Determination of Biochemical Parameter and Oxidative Stress Level

Glucose, TC, TAG, HDL-C, LDL-C, HbA₁c, and IL-6 were analyzed by routine hospital methods in the Laboratory of Analysis of the Complejo Hospitalario of Jaén. Particularly, glucose, TC, TAG, and HDL-C were measured by a spectrophotometric procedure using a Cobas c analyzer (Roche/Hitachi). LDL-C levels were estimated indirectly with the Friedewald equation. The percentage of HbA₁c was determined by a turbidimetric inhibition immunoassay (TINIA) of hemolyzed blood samples on a Cobas c-513 analyzer (Roche). IL-6 was quantified by an electrochemiluminescence immunoassay on a Cobas e-801 analyzer (Roche).

### 4.6. Evaluation of Oxidative Stress

The nitric oxide level was indirectly quantified by measuring nitrate/nitrite and S-nitroso compounds using an ozone chemiluminescence-based method adapted to serum samples [74,75] in a NO analyzer (NOA™ 280i Sievers Instruments, Denver, CO, USA). Lipid peroxidation, indicative of oxidative stress, was measured using the method described by Buege and Aust [76].

### 4.7. Proteomic Analysis

Protein was extracted using 7 M urea, 2 M Thiourea, 4% CHAPS, and 5 mM DTT, and then digested following the filter-aided FASP protocol described by Wisniewski et al. [77], with minor modifications. Trypsin was added in a trypsin:protein ratio of 1:20, and the mixture was incubated overnight at 37 °C, dried out in a RVC2 25 speedvac concentrator (Christ), and resuspended in 0.1% FA. Peptides were desalted and resuspended in 0.1% FA using C18 stage tips (Millipore, Burlington, MA, USA).

Samples were analyzed in a timsTOF Pro mass spectrometer with parallel accumulation serial fragmentation (Bruker Daltonics, Billerica, MA, USA), coupled online to a Evosep ONE liquid chromatograph (Evosep, Odense, Demark). Then, 200 ng was directly loaded onto the Evosep ONE and resolved using the 60 samples-per-day protocol.

Protein identification and quantification were carried out using PEAKS Xpro software (Bioinformatics solutions, Waterloo, ON, Canada). Searches were carried out against a database consisting of Homo sapiens entries from Uniprot Swissprot. Precursor and fragment tolerances of 20 ppm and 0.05 Da were considered for the searches, respectively. Only proteins identified with peptides at FDR < 1% were considered for further analysis. Data were loaded onto the Perseus platform for data processing (log2 transformation, selection of proteins identified in at least 70% of the samples of at least one of the groups, and imputation) and statistical analysis (Student’s *t*-test). Proteins with a *p* < 0.05 were considered for further analyses and discussion.

DEP were analyzed using the Canonical Pathways, Diseases, and Functions and Network-building tools of Ingenuity Pathways Analysis (IPA; Ingenuity^®^ Systems, Redwood City, CA, USA, www.ingenuity.com, accessed on 20 April 2023).

### 4.8. Ethics

The protocol was approved on 24 February 2022 by the Research Ethics Committee of Jaén (Spain). All participants signed the informed consent upon explanation of all the objectives and methodology of the research. This study was conducted according to the recommendations of the Helsinki Declaration and the current Spanish directives.

### 4.9. Statistical Analysis

First, a descriptive analysis was performed, including boxplot graphs. Second, the data were analyzed using repeated-measures two-way ANOVA. Group (control and HT), time (0 d, 45 d, and 90 d), and their interaction were the main effects. When significant differences were detected, Tukey’s HSD test was used to determine whether there was a group effect.

To ensure the validity of the results, diagnostics were conducted to test the assumptions of the two-way repeated-measures ANOVA. In cases where these assumptions were not met, non-parametric analyses were employed. Specifically, the Friedmann test was used to examine significant differences in the variables of interest at different time points, while the Mann–Whitney test was used to evaluate significant differences between the two groups under consideration.

All statistical analyses were carried out using the IBM SPSS Statistics 27 and R statistical (version R.4.2.3) software.

## Figures and Tables

**Table 1:** Anthropometric, clinical, and biochemical characteristics of control and HT-treated stroke patients at 0, 45, and 90 days.

	HT Treatment (*n* = 4)			Contro1 (*n* = 4)			*p*-Value	
	0 d	45 d	90 d	0 d	45 d	90 d	Time Effect	Group Effect
Sex	Men			Men				
Age (years)	63.8 ± 9.3			74.8 ± 4.8				
BMI (kg/m^2^)	37.4 ± 8.6			31.5 ± 3.1				
Glucose (mg/dL)	98.8 ± 7.5	115.5 ± 13.2	101.0 ± 4.1	119.3 ± 2.4	115.5 ± 16.4	116.3 ± 17.9		
TAG (mg/dL)	112.5 ± 60.0	92.8 ± 40.7	101.5 ± 37.5	137.0 ± 55.7	121.8 ± 45.3	138.5 ± 83.3		
HDL-C (mg/dL)	44.0 ± 13.5	42.5 ± 9.3	39.3 ± 4.2	41.3 ± 15.2	38.8 ± 2.9	34.3 ± 8.8		
LDL-C (mg/dL)	98.3 ± 45.5	70.8 ± 19.5	56.5 ± 15.6	100.0 ± 29.0	58.0 ± 10.4	47.8 ± 21.8	0.008	
TC (mg/dL)	165.0 ± 48.0	131.8 ± 23.1	116.0 ± 10.6	169.0 ± 39.6	121.3 ± 13.9	109.5 ± 21.8	0.07	
HbA1c (%)	0.06 ± 0.01	0.06 ± 0.01	0.06 ± 0.01	0.06 ± 0.01	0.06 ± 0.01	0.06 ± 0.01		
IL-6 (pg/mL)	9.9 ± 10.7	4.8 ± 4.1	3.3 ± 15	73.6 ± 130.7	13.3 ± 18.4	12.2 ± 14.2		
NO (μM)	4.7 ± 1.9	5.6 ± 0.5	14.7 ± 9.7	6.1 ± 0.3	11.5 ± 2.4	8.0 ± 2.8		0.010
TBARS (μM)	7.8 ± 1.2	10.4 ± 3.1	8.8 ± 2.6	7.0 ± 2.5	8.8 ± 2.4	11.4 ± 3.3	0.063	
SBP (mmHg)	144.8 ± 34.8	128.8 ± 8.7	118.8 ± 10.3	163.3 ± 12.0	121.0 ± 8.4	120.5 ± 7.4	0.004	
DBP (mmHg)	81.5 ± 9.7	72.3 ± 3.2	60.3 ± 21.1	92.5 ± 10.9	67.5 ± 5.0	66.8 ± 10.4	0.001	
NIHSS	5.3 ± 1.5	0.5 ± 1.0	0.25 ± 0.5	8 ± 2.5	2.5 ± 2.7	2.0 ± 2.5	0.001	
mRs	13 ± 1.0	0.0 ± 0.0	0.3 ± 0.5	3.3 ± 15	1.5 ± 1.3	1.3 ± 1.0	<0.001	
TUG (s)	–	12.8 ± 2.9	13.6 ± 6.9	–	14.3 ± 5.5	11.5 ± 2.7		
FAC	–	3.75 ± 1.0	3.5 ± 2.4	–	4 ± 1.4	4 ± 1.4		
HGS non-paretic limb (kg)	–	34.1 ± 4.3	34.3 ± 7.2	–	27.2 ± 3.0	29.4 ± 9.76		
HGS paretic limb (kg)	–	22.9 ± 8.0	24.65 ± 9.7	–	14.6± 11.0	16.4 ± 13.2		
MoCA score	–	24.8 ± 5.0	24.5 ± 8.0	–	20 ± 8.7	24.3 ± 6.0		

Values are expressed as mean ± SD. Only statistically (*p* < 0.05) or marginally (0.1 > *p* > 0.05) significant differences are shown. BMI: body mass index, DBP: diastolic blood pressure, FAC: Functional Ambulation Categories, HbA₁c: glycated hemoglobin, HDL-C: high-density lipoprotein cholesterol, HGS: hand-grip strength, IL-6: interleukin 6, LDL-C: low-density lipoprotein cholesterol, MoCA score: Montreal Cognitive Assessment score, mRs: modification of Rankin Scale, NIHSS: National Institutes of Health Stroke Scale, NO: nitric oxide, SBP: systolic blood pressure, TAG: triacylglycerides, TBARS: thiobarbituric acid-reactive substances, TC: total cholesterol, TUG: Timed Up and Go.

**Table 2:** Proteins differentially expressed in the serum of control and HT-treated stroke patients.

HT Treatment					
45 vs. 0					
Uniprot ID	Protein Name	Abbreviation	Gene	Fold Change	*p* Value
000533	Neural cell adhesion molecule L1-like protein	NC HL1	CHL1	1.912	0.026
Q9UGM5	Fetuin-B	FETUB	FETUB	1.359	0.045
P07339	Cathepsin D	CATD	CTSD	−1.169	0.012
P04114	Apolipoprotein B-100	APOB	APOB	−1.655	0.014
Q9UHG3	Prenylcysteine oxidase	PCYOX	PCYOX1	−1.716	0.030
P02649	Apolipoprotein E	APOE	APOE	−1.891	0.000
O95445	Apolipoprotein M	APOM	APOM	−1.968	0.039
Q13740	CD166 antigen	CD166	ALCAM	−2.192	0.039
Q005469	Hormone- sensitive lipase	LIPS	LIPE	−3.229	0.041
90 vs. 45					
Uniprot ID	PrSSotein name	Abbreviation	Gene	Fold change	*p* value
P02766v109B	Transthyretinv109B	TTR	TTR	6.972	0.048
Q8N1N4	Keratin type I cytoskeletal 78	K2C78	KRT78	5.137	0.043
90 vs. 0					
Uniprot ID	Protein name	Abbreviation	Gene	Fold change	*p* value
P55065	Apolipoprotein A-IV	APC )A4	APOA4	2.987	0.008
P06727	Glutaminyl-peptide cydotransferase	QPCT	QPCT	2.189	0.023
Q16769	Keratin type I cytoskeletal 14	K1C14	KRT14	1.704	0.031
P00558	Fetuin-B	FETUB	FETUB	1.554	0.038
P02533	Hyaluronan-binding protein 2	HABP2	HABP2	1.44	0.037
Q14520	Phosphoglycerate kinase	PGK1	PGK1	−1.268	0.030
Q9UGM5	Apolipoprotein M	APC DM	APC DM	−1.927	0.043
P02654	Apolipoprotein C-I	APOC 1	APOC 1	−2.624	0.042
O95445	Apolipoprotein C-IV	APOC 4	APOC4	−6.702	0.006
CONTROL					
45 vs. 0					
Uniprot ID	Protein name	Abbreviation	Gene	Fold change	*p* value
P06727	Apolipoprotein A-IV	APOA4	APOA4	2.903	0.032
O43505	Beta-1 4-glucuronyltransferase 1	B4GA1	B4GAT1	2.274	0.002
Q12860	Contactin-1	CNTN1	CNTN1	2.107	0.037
P01008	Antithrombin-III	ANT3	SERPINC1	1.495	0.040
P06681	Complement C2	CO2	C2	1.138	0.034
P04180	Phosphatidylcholine -sterol acyltransferase	LCAT	LCAT	−1.389	0.023
P22352	Glutathione peroxidase 3	GPX3	GPX3	−1.572	0.042
P04066	Tissue alpha-L-fucosidase	FUCO	FUC A1	−3.751	0.007
90 vs. 45					
Uniprot ID	Protein name	Abbreviation	Gene	Fold change	*p* value
Q6YHK3	CD109 antigen	CD109	CD109	3.72	0.034
O14818	Proteasome subunit alpha type-7	PSA7	PSMA7	1.494	0.028
P35542	Serum amyloid A-4 protein	SAA4	SAA4	−1.404	0.030
Q5TZA2	Rootletin	CROCC	CROCC	−1.454	0.042
O43505	Beta-1 4-glucuronyltransferase 1	B4GA1	B4GAT1	−2.695	0.029
P00740	Coagulation factor IX	FA9	F9	−3.156	0.024
P16157	Ankyrin-1	ANK1	ANK1	−9.475	0.022
90 vs. 0					
Uniprot ID	Protein name	Abbreviation	Gene	Fold change	*p* value
Q6YHK3	CD109 antigen	CD109	CD109	8.496	0.016
A0A075B6H7	Probable non-functional immunoglobulin kappa variable 3-7	KV37	IGKV3	7.943	0.044
Q12860	Contactin-1	CNTN1	CNTN1	6.574	0.020
P06727	Apolipoprotein A-IV	APOA4	APC )A4	3.089	0.013
P00352	Retinal dehydrogenase 1	AL1A1	ALDH1A1	1.911	0.042
P01008	Antithrombin-III	ANT3	SERPINC1	1.46	0.014
P35542	Serum amyloid A-4	SAA4	SAA4	−1.805	0.025
P02652	Apolipoprotein A-II	APOA2	APOA2	−1.991	0.050
P01591	Immunoglobulin J chain	IGJ	JCHAIN	−2.23	0.025
P02654	Apolipoprotein C-I	APOC1	APOC1	−3.188	0.037
P00740	Coagulation factor IX	FA9	F9	−3.298	0.035
A0A0C4DH67	Immunoglobulin kappa variable 1-8	KV108	IGKV1	−3.952	0.018
O95445	Apolipoprotein M	APOM	APOM	−3.984	0.030
P04066	Tissue alpha-L-fucosidase	FUCO	FUCA1	−8.264	0.035
Q03591	Complement factor H-related protein 1	FHR1	CFHR1	−26.803	0.050

## Data Availability

The raw data supporting the conclusions of this article will be made available by the authors on request.

## References

[1] Feigin V.L., Brainin M., Norrving B., Martins S., Sacco R.L., Hacke W., Fisher M., Pandian J., Lindsay P. (2022). World Stroke Organization (WSO): Global Stroke Fact Sheet 2022. Int. J. Stroke.

[2] Informe Anual del Sistema Nacional de Salud 2020–2021. https://www.sanidad.gob.es/estadEstudios/estadisticas/sisInfSanSNS/tablasEstadisticas/InfAnualSNS2022/INFORME_ANUAL_2022.pdf.

[3] Pandya R.S., Mao L., Zhou H., Zhou S., Zeng J., Popp A.J., Wang X. (2011). Central nervous system agents for ischemic stroke: Neuroprotection mechanisms. Cent. Nerv. Syst. Agents Med. Chem..

[4] Beslow L.A., Smith S.E., Vossough A., Licht D.J., Kasner S.E., Favilla C.G., Halperin A.R., Gordon D.M., Jones C.I., Cucchiara A.J. (2011). Hemorrhagic transformation of childhood arterial ischemic stroke. Stroke.

[5] O’Donnell M.J., Xavier D., Liu L., Zhang H., Chin S.L., Rao-Melacini P., Rangarajan S., Islam S., Pais P., McQueen M.J. (2010). Risk factors for ischaemic and intracerebral haemorrhagic stroke in 22 countries (the INTERSTROKE study): A case-control study. Lancet.

[6] Georgakis M.K., Harshfield E.L., Malik R., Franceschini N., Langenberg C., Wareham N.J., Markus H.S., Dichgans M. (2021). Diabetes Mellitus, Glycemic Traits, and Cerebrovascular Disease: A Mendelian Randomization Study. Neurology.

[7] Gardener H., Wright C.B., Gu Y., Demmer R.T., Boden-Albala B., Elkind M.S., Sacco R.L., Scarmeas N. (2011). Mediterranean-style diet and risk of ischemic stroke, myocardial infarction, and vascular death: The Northern Manhattan Study. Am. J. Clin. Nutr..

[8] Aparicio-Soto M., Sanchez-Hidalgo M., Cardeno A., Rosillo M.A., Sanchez-Fidalgo S., Utrilla J., Martin-Lacave I., Alarcon-de-la-Lastra C. (2016). Dietary extra virgin olive oil attenuates kidney injury in pristane-induced SLE model via activation of HO-1/Nrf-2 antioxidant pathway and suppression of JAK/STAT, NF-kappaB and MAPK activation. J. Nutr. Biochem..

[9] Visioli F., Poli A., Gall C. (2002). Antioxidant and other biological activities of phenols from olives and olive oil. Med. Res. Rev..

[10] Bayram B., Ozcelik B., Grimm S., Roeder T., Schrader C., Ernst I.M., Wagner A.E., Grune T., Frank J., Rimbach G. (2012). A diet rich in olive oil phenolics reduces oxidative stress in the heart of SAMP8 mice by induction of Nrf2-dependent gene expression. Rejuvenation Res..

[11] George P.M., Steinberg G.K. (2015). Novel Stroke Therapeutics: Unraveling Stroke Pathophysiology and Its Impact on Clinical Treatments. Neuron.

[12] Campbell B.C.V., De Silva D.A., Macleod M.R., Coutts S.B., Schwamm L.H., Davis S.M., Donnan G.A. (2019). Ischaemic stroke. Nat. Rev. Dis. Primers.

[13] Gonzalez-Correa J.A., Navas M.D., Lopez-Villodres J.A., Trujillo M., Espartero J.L., De La Cruz J.P. (2008). Neuroprotective effect of hydroxytyrosol and hydroxytyrosol acetate in rat brain slices subjected to hypoxia-reoxygenation. Neurosci. Lett..

[14] Cabrerizo S., De La Cruz J.P., Lopez-Villodres J.A., Munoz-Marin J., Guerrero A., Reyes J.J., Labajos M.T., Gonzalez-Correa J.A. (2013). Role of the inhibition of oxidative stress and inflammatory mediators in the neuroprotective effects of hydroxytyrosol in rat brain slices subjected to hypoxia reoxygenation. J. Nutr. Biochem..

[15] De La Cruz J.P., Ruiz-Moreno M.I., Guerrero A., Reyes J.J., Benitez-Guerrero A., Espartero J.L., Gonzalez-Correa J.A. (2015). Differences in the Neuroprotective Effect of Orally Administered Virgin Olive Oil (*Olea europaea*) Polyphenols Tyrosol and Hydroxytyrosol in Rats. J. Agric. Food Chem..

[16] De La Cruz J.P., Ruiz-Moreno M.I., Guerrero A., Lopez-Villodres J.A., Reyes J.J., Espartero J.L., Labajos M.T., Gonzalez-Correa J.A. (2015). Role of the catechol group in the antioxidant and neuroprotective effects of virgin olive oil components in rat brain. J. Nutr. Biochem..

[17] Reyes J.J., Villanueva B., Lopez-Villodres J.A., De La Cruz J.P., Romero L., Rodriguez-Perez M.D., Rodriguez-Gutierrez G., Fernandez-Bolanos J., Gonzalez-Correa J.A. (2017). Neuroprotective Effect of Hydroxytyrosol in Experimental Diabetes Mellitus. J. Agric. Food Chem..

[18] Calahorra J., Shenk J., Wielenga V.H., Verweij V., Geenen B., Dederen P.J., Peinado M.A., Siles E., Wiesmann M., Kiliaan A.J. (2019). Hydroxytyrosol, the Major Phenolic Compound of Olive Oil, as an Acute Therapeutic Strategy after Ischemic Stroke. Nutrients.

[19] Barca C., Wiesmann M., Calahorra J., Wachsmuth L., Doring C., Foray C., Heiradi A., Hermann S., Peinado M.A., Siles E. (2021). Impact of hydroxytyrosol on stroke: Tracking therapy response on neuroinflammation and cerebrovascular parameters using PET-MR imaging and on functional outcomes. Theranostics.

[20] Hochrainer K., Yang W. (2022). Stroke Proteomics: From Discovery to Diagnostic and Therapeutic Applications. Circ. Res..

[21] Brea D., Rodriguez-Gonzalez R., Sobrino T., Rodriguez-Yanez M., Blanco M., Castillo J. (2011). Proteomic analysis shows differential protein expression in endothelial progenitor cells between healthy subjects and ischemic stroke patients. Neurol. Res..

[22] Boehme A.K., McClure L.A., Zhang Y., Luna J.M., Del Brutto O.H., Benavente O.R., Elkind M.S. (2016). Inflammatory Markers and Outcomes after Lacunar Stroke: Levels of Inflammatory Markers in Treatment of Stroke Study. Stroke.

[23] Katsanos A.H., Malhotra K., Ahmed N., Seitidis G., Mistry E.A., Mavridis D., Kim J.T., Veroniki A.A., Maier I., Matusevicius M. (2022). Blood Pressure after Endovascular Thrombectomy and Outcomes in Patients with Acute Ischemic Stroke: An Individual Patient Data Meta-analysis. Neurology.

[24] Diprose W.K., Wang M.T.M., McFetridge A., Sutcliffe J., Barber P.A. (2020). Glycated hemoglobin (HbA1c) and outcome following endovascular thrombectomy for ischemic stroke. J. Neurointerv. Surg..

[25] Santangelo C., Filesi C., Vari R., Scazzocchio B., Filardi T., Fogliano V., D’Archivio M., Giovannini C., Lenzi A., Morano S. (2016). Consumption of extra-virgin olive oil rich in phenolic compounds improves metabolic control in patients with type 2 diabetes mellitus: A possible involvement of reduced levels of circulating visfatin. J. Endocrinol. Investig..

[26] Binou P., Stergiou A., Kosta O., Tentolouris N., Karathanos V.T. (2023). Positive contribution of hydroxytyrosol-enriched wheat bread to HbA(1)c levels, lipid profile, markers of inflammation and body weight in subjects with overweight/obesity and type 2 diabetes mellitus. Eur. J. Nutr..

[27] Flint A.C., Conell C., Ren X., Banki N.M., Chan S.L., Rao V.A., Melles R.B., Bhatt D.L. (2019). Effect of Systolic and Diastolic Blood Pressure on Cardiovascular Outcomes. N. Engl. J. Med..

[28] Ikonomidis I., Katogiannis K., Chania C., Iakovis N., Tsoumani M., Christodoulou A., Brinia E., Pavlidis G., Thymis J., Tsilivarakis D. (2023). Association of hydroxytyrosol enriched olive oil with vascular function in chronic coronary disease. Eur. J. Clin. Investig..

[29] Hara T., Fukuda D., Ganbaatar B., Pham P.T., Aini K., Rahadian A., Suto K., Yagi S., Kusunose K., Yamada H. (2023). Olive mill wastewater and hydroxytyrosol inhibits atherogenesis in apolipoprotein E-deficient mice. Heart Vessel..

[30] Hennekens C.H., Gaziano J.M. (1993). Antioxidants and heart disease: Epidemiology and clinical evidence. Clin. Cardiol..

[31] Narne P., Pandey V., Phanithi P.B. (2019). Role of Nitric Oxide and Hydrogen Sulfide in Ischemic Stroke and the Emergent Epigenetic Underpinnings. Mol. Neurobiol..

[32] Paspalj D., Nikic P., Savic M., Djuric D., Simanic I., Zivkovic V., Jeremic N., Srejovic I., Jakovljevic V. (2015). Redox status in acute ischemic stroke: Correlation with clinical outcome. Mol. Cell. Biochem..

[33] Serrano-Ponz M., Rodrigo-Gasque C., Siles E., Martinez-Lara E., Ochoa-Callejero L., Martinez A. (2016). Temporal profiles of blood pressure, circulating nitric oxide, and adrenomedullin as predictors of clinical outcome in acute ischemic stroke patients. Mol. Med. Rep..

[34] Calahorra J., Martinez-Lara E., De Dios C., Siles E. (2018). Hypoxia modulates the antioxidant effect of hydroxytyrosol in MCF-7 breast cancer cells. PLoS ONE.

[35] Yuan S., Tang B., Zheng J., Larsson S.C. (2020). Circulating Lipoprotein Lipids, Apolipoproteins and Ischemic Stroke. Ann. Neurol..

[36] Banfi C., Baetta R., Barbieri S.S., Brioschi M., Guarino A., Ghilardi S., Sandrini L., Eligini S., Polvani G., Bergman O. (2021). Prenylcysteine oxidase 1, an emerging player in atherosclerosis. Commun. Biol..

[37] Plubell D.L., Fenton A.M., Rosario S., Bergstrom P., Wilmarth P.A., Clark W.M., Zakai N.A., Quinn J.F., Minnier J., Alkayed N.J. (2020). High-Density Lipoprotein Carries Markers That Track with Recovery from Stroke. Circ. Res..

[38] Sacks F.M., Alaupovic P., Moye L.A., Cole T.G., Sussex B., Stampfer M.J., Pfeffer M.A., Braunwald E. (2000). VLDL, apolipoproteins B, CIII, and E, and risk of recurrent coronary events in the Cholesterol and Recurrent Events (CARE) trial. Circulation.

[39] Sidorov E.V., Xu C., Garcia-Ramiu J., Blair A., Ortiz-Garcia J., Gordon D., Chainakul J., Sanghera D.K. (2022). Global Metabolomic Profiling Reveals Disrupted Lipid and Amino Acid Metabolism Between the Acute and Chronic Stages of Ischemic Stroke. J. Stroke Cerebrovasc. Dis..

[40] Escary J.L., Choy H.A., Reue K., Wang X.P., Castellani L.W., Glass C.K., Lusis A.J., Schotz M.C. (1999). Paradoxical effect on atherosclerosis of hormone-sensitive lipase overexpression in macrophages. J. Lipid Res..

[41] Qu J., Ko C.W., Tso P., Bhargava A. (2019). Apolipoprotein A-IV: A Multifunctional Protein Involved in Protection against Atherosclerosis and Diabetes. Cells.

[42] van Lierop Z.Y., Noteboom S., Steenwijk M.D., van Dam M., Toorop A.A., van Kempen Z.L., Moraal B., Barkhof F., Uitdehaag B.M., Schoonheim M.M. (2022). Neurofilament-light and contactin-1 association with long-term brain atrophy in natalizumab-treated relapsing-remitting multiple sclerosis. Mult. Scler..

[43] Zhang M., Yu Q., Tang W., Wu Y., Lv J., Sun L., Shi G., Wu M., Qu J., Di C. (2021). Epithelial exosomal contactin-1 promotes monocyte-derived dendritic cell-dominant T-cell responses in asthma. J. Allergy Clin. Immunol..

[44] Li S.J., Ma M.H., Li J.M., Lu X.Y., Lu C.B., Zhou S.F., Zhang L.X., Li M.Q., Shao T.Z., Bai S.P. (2023). CNTN1 Aggravates Neuroinflammation and Triggers Cognitive Deficits in Male Mice by Boosting Crosstalk between Microglia and Astrocytes. Aging Dis..

[45] Papagiannis A., Gkolfinopoulou C., Tziomalos K., Dedemadi A.G., Polychronopoulos G., Milonas D., Savopoulos C., Hatzitolios A.I., Chroni A. (2023). HDL cholesterol efflux capacity and phospholipid content are associated with the severity of acute ischemic stroke and predict its outcome. Clin. Chim. Acta.

[46] Jin R.C., Mahoney C.E., Coleman Anderson L., Ottaviano F., Croce K., Leopold J.A., Zhang Y.Y., Tang S.S., Handy D.E., Loscalzo J. (2011). Glutathione peroxidase-3 deficiency promotes platelet-dependent thrombosis in vivo. Circulation.

[47] Meng R., Li Z.Y., Ji X., Ding Y., Meng S., Wang X. (2011). Antithrombin III associated with fibrinogen predicts the risk of cerebral ischemic stroke. Clin. Neurol. Neurosurg..

[48] Praissman J.L., Live D.H., Wang S., Ramiah A., Chinoy Z.S., Boons G.J., Moremen K.W., Wells L. (2014). B4GAT1 is the priming enzyme for the LARGE-dependent functional glycosylation of alpha-dystroglycan. eLife.

[49] Zhang X., Yuan H., Lyu J., Meng X., Tian Q., Li Y., Zhang J., Xu X., Su J., Hou H. (2021). Association of dementia with immunoglobulin G N-glycans in a Chinese Han Population. NPJ Aging Mech. Dis..

[50] Gao C., Zhang B., Zhang W., Pu S., Yin J., Gao Q. (2011). Serum prealbumin (transthyretin) predict good outcome in young patients with cerebral infarction. Clin. Exp. Med..

[51] Ambrosius W., Michalak S., Kazmierski R., Andrzejewska N., Kozubski W. (2017). Predictive value of serum transthyretin for outcome in acute ischemic stroke. PLoS ONE.

[52] Zhang S.Q., Peng B., Stary C.M., Jian Z.H., Xiong X.X., Chen Q.X. (2017). Serum prealbumin as an effective prognostic indicator for determining clinical status and prognosis in patients with hemorrhagic stroke. Neural. Regen. Res..

[53] Isono N., Imamura Y., Ohmura K., Ueda N., Kawabata S., Furuse M., Kuroiwa T. (2017). Transthyretin Concentrations in Acute Stroke Patients Predict Convalescent Rehabilitation. J. Stroke Cerebrovasc. Dis..

[54] He J., Zhu J., Zhang W., Zhan Z., Fu F., Bao Q. (2022). Association between serum transthyretin and intracranial atherosclerosis in patients with acute ischemic stroke. Front. Neurol..

[55] Guo Q., Lu T., Xu H., Luo Q., Liu Z., Jiang S., Pan J., Lin S., Lin M., Guo F. (2023). Identification of immune-related genes contributing to head and neck squamous cell carcinoma development using weighted gene co-expression network analysis. Cancer Rep..

[56] Birjmohun R.S., Dallinga-Thie G.M., Kuivenhoven J.A., Stroes E.S., Otvos J.D., Wareham N.J., Luben R., Kastelein J.J., Khaw K.T., Boekholdt S.M. (2007). Apolipoprotein A-II is inversely associated with risk of future coronary artery disease. Circulation.

[57] Prentice R.L., Paczesny S., Aragaki A., Amon L.M., Chen L., Pitteri S.J., McIntosh M., Wang P., Buson Busald T., Hsia J. (2010). Novel proteins associated with risk for coronary heart disease or stroke among postmenopausal women identified by in-depth plasma proteome profiling. Genome Med..

[58] Ribas V., Sanchez-Quesada J.L., Anton R., Camacho M., Julve J., Escola-Gil J.C., Vila L., Ordonez-Llanos J., Blanco-Vaca F. (2004). Human apolipoprotein A-II enrichment displaces paraoxonase from HDL and impairs its antioxidant properties: A new mechanism linking HDL protein composition and antiatherogenic potential. Circ. Res..

[59] Hage V. (2011). The NIH stroke scale: A window into neurological status. Nurs. Spectr..

[60] Samuel O.W., Fang P., Chen S., Geng Y., Li G., Khan S.U., Zomaya A.Y., Abbas A. (2017). Activity recognition based on pattern recognition of myoelectric signals for rehabilitation. Handbook of Large-Scale Distributed Computing in Smart Healthcare.

[61] Dominguez R., Vila J.F., Augustovski F., Irazola V., Castillo P.R., Rotta Escalante R., Brott T.G., Meschia J.F. (2006). Spanish cross-cultural adaptation and validation of the National Institutes of Health Stroke Scale. Mayo Clin. Proc..

[62] Banks J.L., Marotta C.A. (2007). Outcomes validity and reliability of the modified Rankin scale: Implications for stroke clinical trials: A literature review and synthesis. Stroke.

[63] Montgomery G., McPhee J., Paasuke M., Sipila S., Maier A.B., Hogrel J.Y., Degens H. (2020). Determinants of Performance in the Timed Up-and-Go and Six-Minute Walk Tests in Young and Old Healthy Adults. J. Clin. Med..

[64] Rossler R., Rommers N., Kim E.K., Iendra L., Sofios A., Giannouli E., Portegijs E., Rantanen T., Infanger D., Bridenbaugh S. (2023). Timed up-and-go performance is associated with objectively measured life space in patients 3 months after ischemic stroke: A cross-sectional observational study. J. Neurol..

[65] Wade D.T. (1992). Measurement in neurological rehabilitation. Curr. Opin. Neurol. Neurosurg..

[66] Price R., Choy N.L. (2019). Investigating the Relationship of the Functional Gait Assessment to Spatiotemporal Parameters of Gait and Quality of Life in Individuals with Stroke. J. Geriatr. Phys. Ther..

[67] Cinnera A.M., Marrano S., De Bartolo D., Iosa M., Bisirri A., Leone E., Stefani A., Koch G., Ciancarelli I., Paolucci S. (2023). Convergent Validity of the Timed Walking Tests with Functional Ambulatory Category in Subacute Stroke. Brain Sci..

[68] Faria C.D.C.M., Aguiar L.T., Lara E.M., Souza L.A.C.E., Martins J.C., Teixeira-Salmela L.F. (2013). Dynamometry for the assessment of grip, pinch, and trunk strength in subjects with chronic stroke: Reliability and various sources of outcome values. Int. J. Phys. Med. Rehabil..

[69] Bernhardt J., Hayward K.S., Kwakkel G., Ward N.S., Wolf S.L., Borschmann K., Krakauer J.W., Boyd L.A., Carmichael S.T., Corbett D. (2017). Agreed definitions and a shared vision for new standards in stroke recovery research: The Stroke Recovery and Rehabilitation Roundtable taskforce. Int. J. Stroke.

[70] Park J.G., Lee K.W., Kim S.B., Lee J.H., Kim Y.H. (2019). Effect of Decreased Skeletal Muscle Index and Hand Grip Strength on Functional Recovery in Subacute Ambulatory Stroke Patients. Ann. Rehabil. Med..

[71] Ademoyegun A.B., Mbada C.E., Sonuga O.A., Malomo O.E., Fatai W.A., Aghedo I.A. (2022). Does grip strength of the less-affected side of ischemic stroke survivors influences performance of self-care activities?. Bull. Fac. Phys. Ther..

[72] Nasreddine Z.S., Phillips N.A., Bedirian V., Charbonneau S., Whitehead V., Collin I., Cummings J.L., Chertkow H. (2005). The Montreal Cognitive Assessment, MoCA: A brief screening tool for mild cognitive impairment. J. Am. Geriatr. Soc..

[73] Gallego M.L., Ferrándiz M.H., Garriga O.T., Nierga I.P., López-Pousa S., Franch J.V. (2009). Validación del Montreal Cognitive Assessment (MoCA): Test de cribado para el deterioro cognitivo leve. Datos preliminares. Alzheimer. Real. Investig. Demenc..

[74] Lopez-Ramos J.C., Martinez-Romero R., Molina F., Canuelo A., Martinez-Lara E., Siles E., Peinado M.A. (2005). Evidence of a decrease in nitric oxide-storage molecules following acute hypoxia and/or hypobaria, by means of chemiluminescence analysis. Nitric. Oxide.

[75] Peinado M.A., Lopez-Ramos J.C., Camacho M.V., Molina F.J., Martinez-Romero R., Hernandez R., Siles E., Martinez-Lara E., Del Moral M.L., Pedrosa J.A. (2007). Age and sex-related serum changes in nitric oxide: Correlations with serological markers. Int. J. Cardiol..

[76] Buege J.A., Aust S.D. (1978). Microsomal lipid peroxidation. Methods Enzymol..

[77] Wisniewski J.R., Zougman A., Nagaraj N., Mann M. (2009). Universal sample preparation method for proteome analysis. Nat. Methods.

